# Unexpected Small Urinary Bladder Pheochromocytoma: A Nonspecific Presentation

**DOI:** 10.1155/2013/496547

**Published:** 2013-09-22

**Authors:** Faouzi Mallat, Wissem Hmida, Adel Slama, Faouzi Mosbah

**Affiliations:** Department of Urology, Hospital of Sahloul, 4054 Sousse, Tunisia

## Abstract

*Objectives*. Pheochromocytoma of the urinary bladder is an extremely rare tumor that typically presents with a hypertensive crisis during micturition. Preoperatively, it may be misdiagnosed due to nonspecific symptomatology, physical, and radiologic findings. *Method*. We report a case of unsuspected small pheochromocytoma which was incidentally found by CT scan and confirmed by the histological aspects after transurethral resection in a 63-year-old woman. Here, we have described the clinical presentation, physical findings, laboratory investigations, and treatment provided in our case. We have also included radiological images and histopathology slides with input from both radiologists and pathologists. Surgical management and postoperative follow-up are discussed, as are details of previous published data. *Results*. After undergoing surgical treatment (transurethral resection), our patient is asymptomatic, with complete resolution of her pathology. *Conclusion*. Diagnosis is difficult before histopathological examination and should be considered in patients with no risk factors for usual bladder tumor. Our purpose is to raise clinician's awareness for this condition so that they will be more likely to diagnose it. This will facilitate prompt diagnosis and treatment and especially prevent complications due to pheochromocytoma which may be severe.

## 1. Introduction

Pheochromocytoma is neuroendocrine tumour that usually develops ahead of the chromaffin cells of the adrenal medulla or para-aortic nerve trunk. Sometimes, it is found at other sites. When its location is in the urinary bladder, the symptoms due to raised catecholamines (headache, blurred vision, heart palpitation, and flushing) may be induced by voiding [1]. However, 27% of pheochromocytomas of the urinary bladder do not feature any hormonal activity [2]. 

We reported an observation of small pheochromocytoma of the urinary bladder discovered incidentally. With a review of the literature the clinical symptoms, diagnosis, and treatment of bladder pheochromocytomas are studied. 

## 2. Case Presentation

A 63-year-old woman having a history of breast cancer treated in 2009 by mastectomy, axillary dissection, radiotherapy and adjuvant chemotherapy was admitted for endoscopic resection of a bladder tumor discovered incidentally on computed tomography (CT) done during a review of its control of breast cancer. 

The patient did not present any history of haematuria or other signs. CT, performed nine months before admission, was normal. 

**Figure 1:**
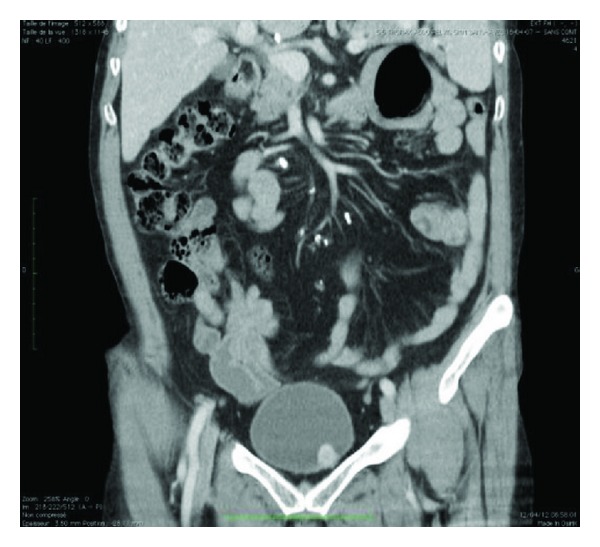
CT scan showing small tumour located in the anterior front of the bladder with well-defined bounds and increasing its density after the injection of iodine-containing contrast solution.

Computed tomography performed one month before admission showed a well-defined 1,8 cm size tumor located in the anterior front of the bladder, with a heterogeneous increase in density after injection of iodine-containing contrast solution (Figure 1). 

On admission, blood pressure was 120/60 mmHg; pulse was 80/minutes and regular. The rest of the physical examination was unremarkable. Laboratory tests and an electrocardiogram were normal. 

Cystoscopy showed small tumor of the bladder dome of 2 cm, not having the usual appearance of urothelial tumor; therefore, the tumor's surface was smooth, and neither ulceration nor active bleeding on the tumor was found. The rest of the bladder was normal. A complete resection of the tumor was performed. 

Given the history of breast cancer and unusual cystoscopic appearance of the tumor, a bladder metastasis of breast cancer was suspected. 

**Figure 2:**
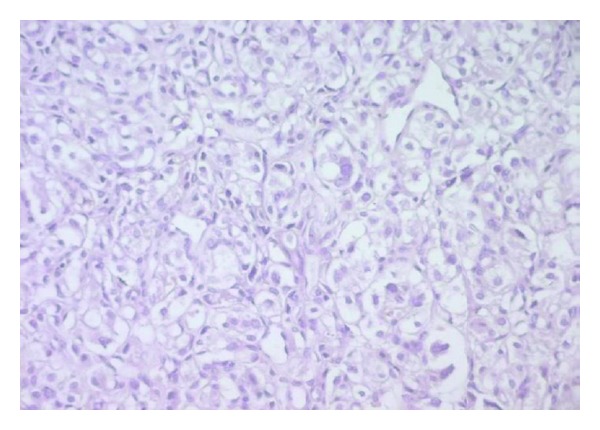
(HEx200) Tumor cells arranged in nests and trabecular patterns surrounded by an endocrine stroma.

**Figure 3:**
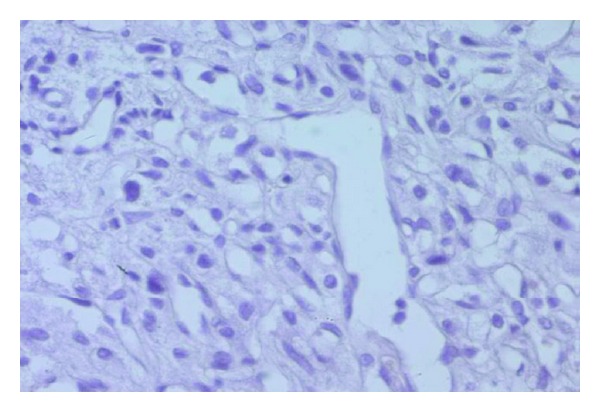
(HEx400) cells with round centrally nuclei with abundant clear cytoplasm.

**Figure 4:**
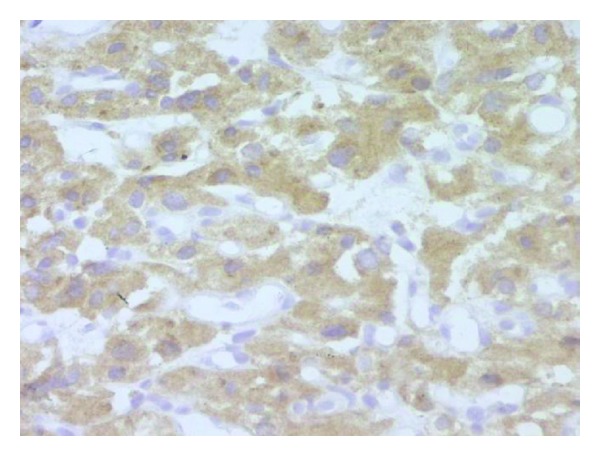
(Chromox400) strong and diffuse cytoplasmic positivity.

**Figure 5:**
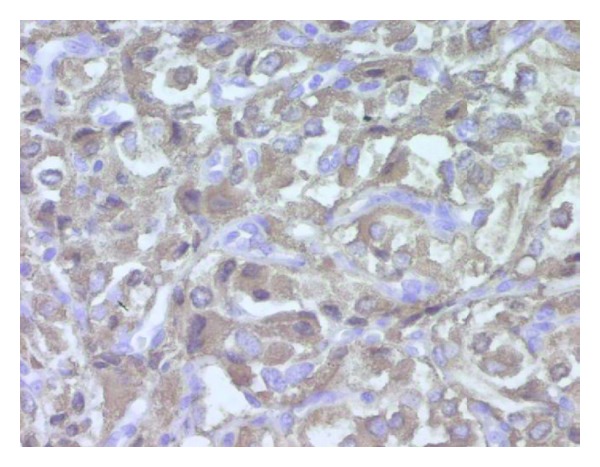
(Synaptox400) the cells show a strong cytoplasmic positivity.

Histological examination of the tumor disclosed typical findings of pheochromocytoma. Microscopically, the tumor was composed of round or polygonal cells arranged in characteristic compact cell nests (Zellballen) or trabecular patterns bound by a delicate fibrovascularstroma (Figure 2). The cells had centrally, round located nuclei with finely clumped chromatin and abundant amount of clear or eosinophilic, granular cytoplasm (Figure 3). There was no cytoplasmatic inclusion, pleomorphism, cytological alterations, or necrosis. The mitotic index was very low. Immunohistochemical studies showed that the cells were strongly reactive for chromogranine (Figure 4), synaptophysin (Figure 5), and NSE (neuron-specific enolase). There was no staining for keratin markers (CK7 and CK20). 

Retrospectively, the patient's past history was reviewed; she had never experienced hypertension, postural hypotension, palpitations, or sudden-onset blurred vision. Intraoperatively, no change in blood pressure or heart rate was noted. During hospitalization, she did not suffer from paroxysmal hypertension. Dosage of metanephrine and normetanephrine 24-hour urine, 4 days after operation, was normal. The course of hospitalization was smooth, and the patient was discharged 9 days after the operation. 

Follow-up cystoscopy performed 3 month later showed no tumor recurrence. The urinary vanillymandelic acid level was 4.0 (normal range, 1.0–11.0) mg/day, and the 131I-MIBG scan was negative at the 6-month follow-up. 

## 3. Discussion 

Pheochromocytoma is a rare catecholamine-producing chromaffin cell tumor [3]. Its incidence is less than 0.06% of all bladder tumors and less than 1% of all pheochromocytomas [1]. It occurs in patients at any age, but the age of peak incidence is between the third and fifth decades; there is no sex predominance in opposition to the adrenal pheochromocytoma that is affecting the female gender in bigger proportion [4]. 

Several case reports of bladder pheochromocytomas were reviewed, and most of them are hormonally active and cause symptoms including palpitations, sweating, headache, and hypertension (paroxysmal/sustained). These symptoms are usually precipitated by bladder contraction during micturition [5]. A rise in blood pressure can be demonstrated immediately following bladder voiding and may be triggered by micturition, defecation, sexual activity, ejaculation, or bladder instrumentation. Haematuria can suggest the diagnosis as 65% of the patients were concerned. These evocative symptoms are observed in 65% of the cases [6]. In those patients, the bladder pheochromocytoma was larger than 2 cm in diameter [2]. 

The above mentioned clinical table recommends the measurement of catecholamines and catecholamine metabolites (metanephrine and normetanephrine) in plasma and 24-hour urine samples. 

Our patient did not have a past history of haematuria, lower abdominal pain, angina, symptoms of postural hypotension, or palpitations, which might have resulted from the pheochromocytoma. Interestingly, no specific symptomatic episodes were reported after voiding or during the transurethral resection. The clinical table was for a nonsecreting tumor. 

In our patient, the VMA level was checked 4 days after the operation, and was found to be within the normal range. Because the half-life of VMA is 8–35 mmol/day, it is not a reliable marker to prove if the tumor had been functional in this patient, because the VMA level was checked 4 days after tumor excision. 

Bladder pheochromocytomas appear as unique tumors [7]. While CT aid in the anatomical localization of these tumors, look for concomitant locations as well as potential ganglionic or visceral metastasis [6]. The magnetic Resonance Imaging (MRI) is more adapted than CT in the evaluation of these tumors [8]. A new imaging method has been recently developed using iodine-131-metaiodobenzylguanidine (MIBG) and indium-111 pentoctreotide scintigraphy serve as complementary functional diagnostic tools as they have 85–100% sensitivity in localizing these tumors [9, 10]. When a pheochromocytoma is suspected and catecholamine measurements are within a normal range, an MIBG scan should be done [11]. 

Through cystoscopy, pheochromocytomas appear as granulated and lobulated lesions with or without ulceration [12]. Similar observations were made for our patient. 

The treatment of the bladder pheochromocytoma is solely surgical, and the patient preparation is an essential step, covering a preoperative treatment with *α*- and *β*-blocking agents. Transurethral resection is not recommended as it will not remove the entire tumor. Open surgery is required to completely resect these tumors [12]. Laparoscopic tumor resection has also been attempted [13]. 

Our reported case was treated with transurethral resection. No drop in blood pressure was noted during the operation. 

As these tumors are known for recurrence and metastases, these patients will require long-term follow-up after the initial operation [14], with regular biological and clinical examinations. MIBG scan may be necessary during follow-up [11]. 

## 4. Conclusion 

Pheochromocytoma of the bladder is a rare tumor. Its diagnosis is suspected in the presence of paroxysmal hyper blood pressure paired with a bladder tumor. In some cases, it can be incidentally found, and it is very important to suspect it to reduce the complications and mismanagement.
